# Comparison of metrics of neonatal intensive care unit antibiotic use

**DOI:** 10.1017/ice.2025.10233

**Published:** 2025-09

**Authors:** Alvaro Zevallos Barboza, Sagori Mukhopadhyay, Karen Marie Puopolo, Di Shu, Jeffrey S. Gerber, Dustin Daniel Flannery

**Affiliations:** 1 Division of Neonatology, Children’s Hospital of Philadelphia, Philadelphia, PA, USA; 2 Clinical Futures, Children’s Hospital of Philadelphia, Philadelphia, PA, USA; 3 Department of Pediatrics, University of Pennsylvania Perelman School of Medicine, Philadelphia, PA, USA; 4 Department of Biostatistics, Epidemiology and Informatics, University of Pennsylvania Perelman School of Medicine, Philadelphia, PA, USA; 5 Division of Infectious Diseases, Children’s Hospital of Philadelphia, Philadelphia, PA, USA

## Abstract

**Objective:**

To compare temporal trends, variation, and correlations between antibiotic use metrics across U.S. neonatal intensive care units (NICUs) and assess associations with mortality.

**Methods:**

We conducted a retrospective cohort study of 438,156 infants admitted to 272 NICUs from 2017 to 2021 using the Premier Health Database. Antibiotic use rate (AUR), days of therapy (DOT), and antibiotic spectrum index (ASI) per 1,000 patient or therapy days were calculated both cumulatively by year and at the center level. Mixed-effects models adjusted for center-level characteristics were used for all analyses.

**Results:**

All three metrics declined over time: AUR by 16.8%, DOT by 19.0%, and ASI by 2.5%. AUR and DOT were highly correlated (*r* = 0.989, *P* < 0.001), while ASI showed weaker correlations with AUR (*r* = 0.247, *P* < 0.001) and DOT (*r* = 0.338, *P* < 0.001). None were significantly associated with center-level mortality. ASI had the least variability, indicating more uniform antibiotic selection and lower center-level discriminatory value.

**Conclusions:**

DOT and AUR were comparable measures of antibiotic consumption, both showing significant declines. ASI exhibited the least variability, reflecting more consistency in antibiotic selection. The similarity in dispersion and decline between AUR and DOT suggests that neonatal antibiotic exposure is primarily influenced by initiation and discontinuation decisions rather than regimen complexity. Given its ease of calculation, AUR may be the most practical metric for evaluating the impact of antibiotic stewardship interventions at the center level.

## Introduction

Antibiotic use metrics are employed to quantify antibiotic consumption, perform intra-hospital and inter-hospital comparisons, assess changes over time and in response to antibiotic stewardship efforts, and to improve quality of care.^1^ However, there are no uniformly accepted antibiotic use metrics for neonatal intensive care units (NICUs).^2^ An ideal metric or combination of metrics should reflect frequency of use, identify variation, relate to pertinent outcomes, and facilitate standardization of care. Assessing antibiotic utilization is a crucial component of antibiotic stewardship because measurement is the first step to improving use, and the final step in assessing stewardship interventions.^1,3^

Multiple antibiotic use metrics have been proposed in pediatric and adult populations, and studies have described use of these metrics in neonatal settings.^2^ These include, but are not limited to, the antibiotic use rate^4–6^ (AUR), days of therapy^7–12^ (DOT), and, more recently, the antibiotic spectrum index^12–15^ (ASI). AUR is calculated by counting the number of patients administered at least one antibiotic in a given day. AUR reflects the *proportion of infants* administered antibiotics in an individual center. DOT is calculated by counting the number of different antibiotics administered on a given day. DOT provides the *amount of antibiotic administered* in an individual center. ASI is calculated by counting the number of antibiotics administered per day and by assigning a numeric value that reflects the spectrum of organisms killed by each antibiotic. ASI provides an *estimate of total antibiotic impact* in an individual center. However, the relative utility of each metric for NICUs is unclear, and most studies to date have included only one or a small number of centers. Further, the relationships between proposed metrics of NICU antibiotic use and associations between these metrics and pertinent neonatal outcomes are poorly understood.

An improved understanding of metrics of antibiotic use in NICUs would allow hospitals and stakeholders to audit antibiotic prescribing more efficiently and accurately and thus improve the safety and quality of neonatal care. Therefore, this study’s objective was to calculate three metrics for antibiotic use (AUR, DOT, and ASI) both cumulatively by year and at the center level, using a large cohort of NICUs across the US and assess trends over time, center variation, correlation between metrics, and associated outcomes, with the overarching goal of identifying the ideal metric(s) for standardization.

## Methods

### Data source and study population

This retrospective cohort study used the Premier Health Database (PHD) data. This U.S. hospital-based repository includes administrative, healthcare utilization, and financial information from more than 750 hospitals and health systems. We included centers that consistently provided annual data from January 1, 2017, to December 31, 2021, with at least 20 NICU admissions each year. The study was classified as exempt by the institutional review board of Children’s Hospital of Philadelphia, with a waiver of informed consent due to the deidentified nature of the patient and center-level data.

### Study definitions

Admission to the NICU was defined using room and board charges. The birth date was designated as the day of hospital admission since only inborn infants were included. Length of stay in days was determined based on hospital service days, including both admission and discharge days. AUR was defined as the total days with at least one antibiotic administration, which was not necessarily continuous, per 1,000 patient days. DOT was defined as the aggregate antibiotic days per 1,000 patient days, where each unique antibiotic administered on a given day contributed to the total. ASI was defined as the sum of assigned points for each antibiotic per day per 1,000 therapy days; ASI is based on activity against clinically relevant pathogens (range 1–13), with a higher ASI indicating a broader spectrum agent (Appendix; adapted from Gerber *et al* ^13^). Only antibiotics with an assigned enteral or parenteral route were included. For a post hoc analysis, early ASI was defined as aggregate ASI in the first three days after birth, and late ASI was defined as aggregate ASI beyond three days after birth. Additional variables were defined according to the PHD data dictionary. The hospital setting, or population served, was classified as urban or rural based on proximity to a city center. A hospital with teaching status was identified as a facility affiliated with a medical school accredited by either the American Medical Association or the Liaison Committee on Medical Education of the Association of American Medical Colleges. Geographic regions were defined using the U.S. Census Geographic Regions. Very low birth weight (VLBW) was assessed utilizing All Patients Refined Diagnosis Related Groups (APR DRGs) and International Classification of Diseases, Tenth Revision (ICD-10) codes, which indicated a birth weight under 1,500 grams. Gestational age <29 weeks was categorized using ICD-10 codes. Infant disposition was determined using Uniform Billing Form (UB-04) discharge status codes.

### Statistical analysis

We calculated the median metrics of antibiotic use (AUR, DOT, and ASI) aggregated at the center level. To assess changes over time, we calculated the overall relative change between 2017 and 2021, the annual relative change (year-to-year), and the average annual relative change for each metric based on values summed annually. The overall relative change represented the percentage of the absolute change in a metric value from 2017 to 2021 relative to the metric value in 2017. The annual relative change indicated the absolute change in a metric value from one year to the following relative to the metric value in the previous year. The average annual relative change represented the mean of the annual relative changes observed from 2017 to 2021. Additionally, we displayed the metric yearly values in a linear chart to visualize trends over time.

To assess variation across the three metrics, we reported the following statistics: median, 25^th^ percentile, 75^th^ percentile, mean, and standard deviation. To facilitate comparisons across metrics, we calculated the minimum and maximum values using *Z*-scores and the coefficient of variation. These statistics standardize values and evaluate relative variability across the metrics. Coefficients of variation express standard deviation as a percentage of the mean, with larger values indicating greater variation. *Z*-scores measure how many standard deviations a data point is from the mean; large *Z*-scores for minimum and maximum values indicate a wider data range. These statistics were visually supported by a multi-panel figure featuring bar graphs displaying the center-level cumulative values of the three metrics during the study period. We also compared metrics across (a) teaching centers and non-teaching centers, (b) rural centers and urban centers, and (c) geographical regions. In post hoc analysis, we compared cumulative center-level ASI values stratified by administration timing (first three days of hospital admission vs after the first three days), using bar charts and density curves to visualize center-level ASI distributions.

To assess the correlation between metrics, we reported Spearman’s rank correlation coefficient (r), which ranges from –1 to 1. Values closer to –1 indicate a stronger negative relationship, values closer to 1 indicate a stronger positive relationship and a value of 0 indicates no relationship.^16^ We utilized scatter plots to compare metrics and visually identify potential relationship trends, and we created a heatmap to simultaneously evaluate the relationships among the three metrics.

Finally, we evaluated the relationships between each metric and crude center mortality rates using a linear mixed-effects model. This model considered potential center-level confounders and included annual repeated measures. Since we lacked the final disposition status for infants transferred from the birth hospital, we conducted a sensitivity analysis to investigate the associations between each metric and a composite outcome of crude center mortality or transfer rates.

Statistical analyses were conducted using SAS version 9.4. A two-sided *P*-value of less than 0.05 was considered statistically significant.

## Results

Among 272 NICUs and 438,156 infants included in the analysis, 166,871 (38.1%) received at least one antibiotic (Table 1). The median annual admissions per NICU was 217 (113, 401). Most NICUs were in urban regions (83.1%) and non-teaching hospitals (62.1%). Forty-five percent of the included infants were male, 3.5% were born with VLBW, and 0.9% died before discharge (Table 1).

**Table 1.** Center-level and infant-level characteristics

Center characteristic	N = 272
Annual NICU admissions (median, IQR)	217 (113,401)
Total hospital beds^a^ (n, %)	
< 300 beds	119 (43.75%)
≥ 300 beds	153 (56.25%)
Setting (n, %)	
Urban	226 (83.1%)
Rural	46 (16.9%)
Teaching status (n, %)	
Teaching hospital	103 (37.9%)
Non-teaching hospital	169 (62.1%)
Geographic region (n, %)	
Northeast	32 (11.8%)
Midwest	71 (26.1%)
South	127 (46.7%)
West	42 (15.4%)
Death rate (Deaths per 1000 NICU infants) (med, IQR)	2.4 (0, 9.6)
Transfer rate (Transfers per 1000 NICU infants) (med, IQR)	65.1 (34.7, 114.7)
Death or transfer rate (Death or transfers per 1000 NICU infants) (median, IQR)	73.1 (44.9, 118.2)
Infant characteristic	N = 438,156
Sex (n, %)	
Male	198,944 (45.4%)
Female	239,125 (54.6%)
Unknown	87 (0.02%)
Very low birth weight (<1500 g) (n, %)	15,256 (3.5%)
Gestational age <29 weeks	18,315 (4.2%)
Length of stay, days (median, IQR)^b^	5 (3 – 14)
Disposition (n, %)	
Discharged home	410,538 (93.7%)
Died	3,844 (0.9%)
Transferred	23,430 (5.3%)
Other/unknown	344 (0.08%)

Note. g (grams); IQR (interquartile range); NICU (neonatal intensive care unit).

a Total hospital beds reflects the total number of all patient beds at a center; number of NICU beds was not available.

b The length of stay reflects the total duration of the birth hospitalization.

**Figure 1.**
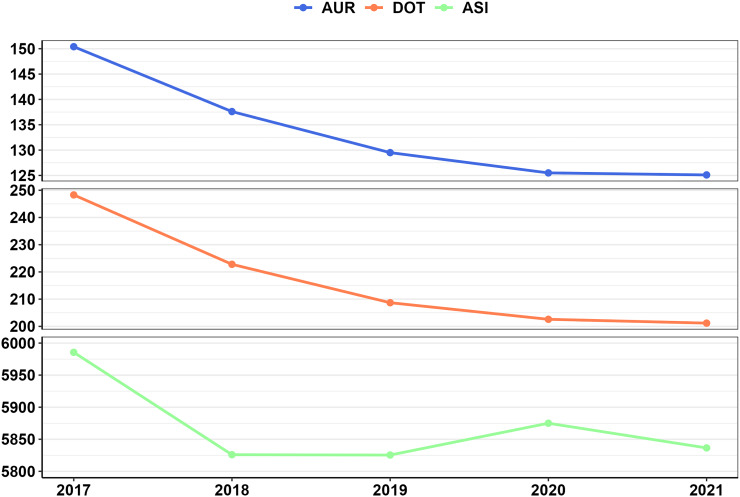
Cumulative annual neonatal antibiotic use metrics over time. *Note*: ASI (antibiotic spectrum index); DOT (days of therapy); AUR (antibiotic use rate). Upper panel: AUR (therapy days per 1000 patient days). Center panel: DOT (antibiotic days per 1000 patient days). Bottom panel: ASI (ASI Score per 1000 therapy days).

Overall median metrics (IQR) were: AUR 124.9 (90.5 – 167.2) per 1000 PD, DOT 208.6 (151.6 – 283.4) per 1000 PD, and ASI 5809.4 (5427.3 – 6049.7) per 1000 PD (Table 2); all displayed a left-skewed distribution. Each metric experienced a relative decline from 2017 to 2021 (AUR by 16.8%; DOT by 18.9%; ASI by 2.5%). However, while AUR and DOT registered an average annual relative decrease of approximately 5% during the study period, ASI had a relative yearly decrease of 0.5% (Table 3). The linear chart further illustrates the difference in annual relative change between AUR and DOT compared to ASI (Figure 1). ASI dropped significantly from 2017 to 2018 and then stabilized, showing only minor variations from 2018 to 2021.

**Table 2.** Variation in center-level neonatal antibiotic use metrics

Metric	Median (IQR)	Min (Z-score)	Max (Z-score)	Mean (SD)	Coefficient of variation^a^
AUR	124.9 (90.5 – 167.2)	17.4 (–1.7)	647.8 (7)	138.8 (73.1)	52.7%
DOT	208.6 (151.6 – 283.4)	30.4 (–1.5)	1210. (7.3)	233.8 (133.0)	56.9%
ASI	5809.4 (5427.3 – 6049.7)	3611.9 (–3.6)	7330.8 (2.7)	5711.2 (590.0)	10.3%

Note. AUR (antibiotic use rate); DOT (days of therapy); antibiotic spectrum index (ASI); IQR (interquartile range); SD (standard deviation) Z-score: statistical measure that shows the relative distance of a data point from the mean of a data set in terms of standard deviations.

a The coefficient of variation ratio was derived from dividing the standard deviation by the mean.

**Table 3.** Cumulative annual neonatal antibiotic use metrics over time

	AUR	DOT	ASI
Overall	133.7	216.8	5872.2
• 2017	150.4	248.3	5985.6
• 2018	137.6	222.8	5825.9
• 2019	129.5	208.7	5825.4
• 2020	125.5	202.6	5875.0
• 2021	125.1	201.2	5836.5
Annual relative change^a^			
• 2017–2018	–8.5%	–10.2%	–2.7%
• 2018–2019	–5.9%	–6.3%	–0.01%
• 2019–2020	–3.1%	–2.9%	0.9%
• 2020–2021	–0.3%	–0.7%	–0.7%
Average annual relative change^b^	–4.5%	–5.1%	–0.6%
Overall relative change^c^			
• 2017–2021	–16.8%	–19.0%	–2.5%

Note. AUR (antibiotic use rate); DOT (days of therapy); antibiotic spectrum index (ASI).

a **Annual relative change**: Proportion that expresses the annual absolute change on a metric value as a percentage of the metric value in the previous year (eg, change in AUR from 2017 to 2018, as a percentage of the AUR value in 2017).

b **Average annual relative change**: Mean value of the annual relative change observed from 2017 to 2021.

c **Overall relative change**: Proportion that expresses the absolute change in a metric value from the first to the last year of the study period, as a percentage of the metric value in the initial year.

**Figure 2.**
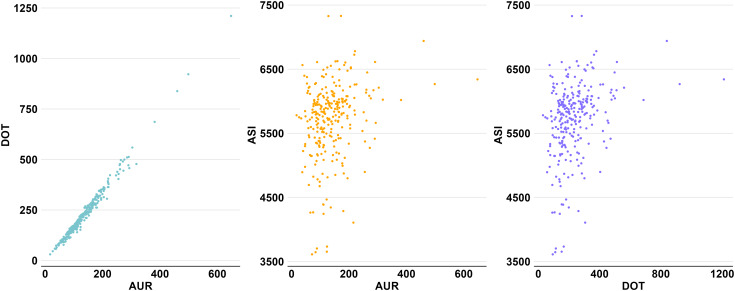
Comparative distribution of center-level neonatal antibiotic use metric values. *Note*: ASI (antibiotic spectrum index); DOT (days of therapy); AUR (antibiotic use rate). Figures were created using R version 4.2.3 to compare the distribution among center-level metric values, calculated using the NICU encounter data from all years of the study period (2017–2021).

The coefficients of variation for the metrics were as follows: AUR (52.7%), DOT (56.9%), and ASI (10.3%). The *Z*-scores for the minimum and maximum values were AUR (–1.7, 7), DOT (–1.5, 7.3), and ASI (–3.6, 2.7) (Table 2). The bar charts indicated left skewness across the three metrics, although ASI exhibited less variation than AUR and DOT (Figure 2). ASI was higher in teaching centers than in non-teaching centers, whereas AUR and DOT showed similarities (Supplemental Table 1). There was no significant difference between rural and urban centers for any of the three (Supplemental Table 1). ASI was lower among centers in the West than other geographic regions (*P* = 0.04), while AUR and DOT were comparable across all regions (Supplemental Table 1).

**Figure 3.**
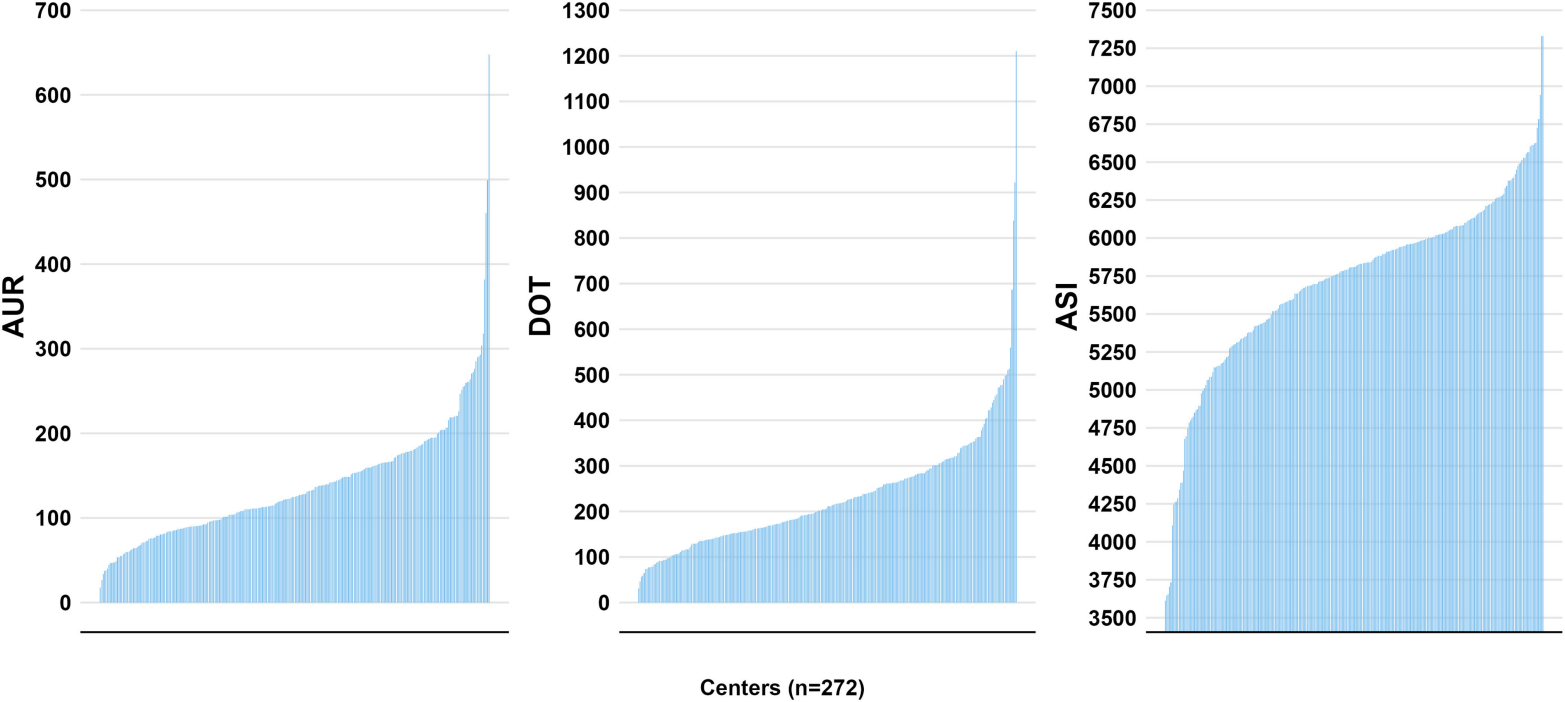
Correlation of center-level neonatal antibiotic use metrics. *Note:* ASI (antibiotic spectrum index); DOT (days of therapy); AUR (antibiotic use rate). Figures were created using R version 4.2.3 and report the cumulative metric values at the center level; each data point represents the respective metric value of a NICU calculated from all admissions from 2017 to 2021. AUR versus DOT (*r* = 0.991; *P* < 0.001); AUR versus ASI (*r* = 0.239; *P* < 0.001); DOT versus ASI (*r* = 0.313; *P* < 0.001).

**Figure 4.**
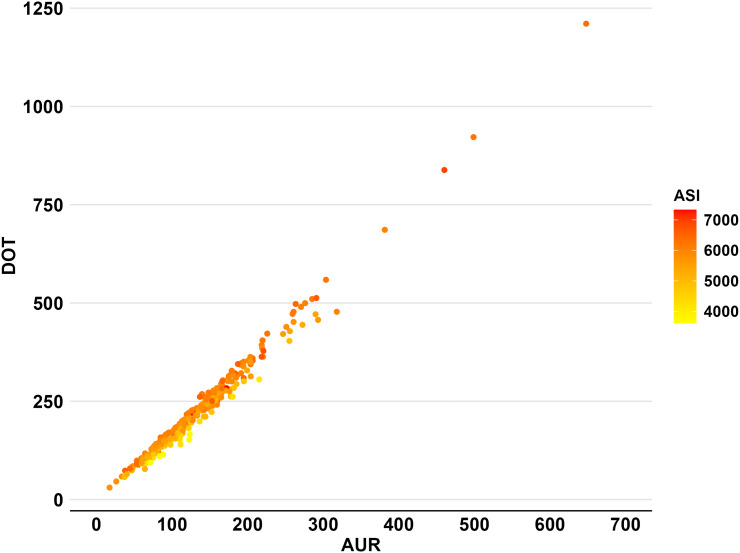
Heat map correlation of three NICU antibiotic use metrics at the center level. *Note:* ASI (antibiotic spectrum index); DOT (days of therapy); AUR (antibiotic use rate). Figures were created using R version 4.2.3 to illustrate the simultaneous correlation among the three metrics. *x*-axis represents the center-level AUR value range, and the *y*-axis the DOT value range. Lastly, the ASI value range is displayed as a color gradient from yellow to red for each data point.

While AUR and DOT were highly correlated (*r* = 0.989 (0.983 – 0.99); *P* < 0.001), ASI exhibited a weak correlation with both AUR (*r* = 0.247 (0.132 – 0.356); *P* < 0.001) and DOT (*r* = 0.338 (0.228 – 0.439); *P* < 0.001). The scatterplots revealed a strong positive linear correlation between AUR and DOT (Figure 3). In contrast, ASI showed only a slight positive linear correlation with both metrics, accompanied by significant data dispersion. These visual findings were further supported by the heat map, where AUR and DOT increased proportionally along the axes, but the color gradient of ASI differed from the other two metrics (Figure 4).

After adjusting for center-level factors, including geographical location, setting (urban or rural), teaching status, the total number of hospital beds, and year, center-level DOT, AUR, and ASI were not significantly associated with center mortality rates (Supplemental Table 2). Sensitivity analyses showed that all three metrics exhibited significant associations with the composite outcome of center death or transfer rates after accounting for the covariates (Supplemental Table 2).

In the post hoc stratified analysis, early center-level ASI exhibited a narrower distribution of ASI values with a higher central tendency. In contrast, the late center-level ASI demonstrated a wider spread and a slightly lower central tendency, yet it encompassed ASI values that exceeded the range observed with early ASI (Supplemental Figure). A Mann-Whitney *U* test confirmed that the median center-level ASI significantly differed between the two groups (*P* < 0.001).

## Discussion

In this center-level study of 438,156 infants admitted to 272 NICUs from 2017 to 2021, we directly evaluated antibiotic use trends over time and the relationships between two commonly used neonatal antibiotic use metrics (AUR and DOT) and a relatively new metric (ASI). All three neonatal antibiotic use metric values decreased over the study period, although at different rates. The ASI rate of decrease was approximately one-tenth that of the other two antibiotic use metrics. AUR and DOT demonstrated similar measures of dispersion, indicating comparable variability across centers, while ASI exhibited less dispersion, signifying less variation across centers. AUR and DOT’s high correlation reinforces their redundancy in reflecting antibiotic use patterns. In contrast, ASI’s poor correlation with AUR and DOT suggests its independence from these more commonly used metrics and its potential as a complementary metric. Finally, all three metrics were not associated with mortality rates but were associated with the composite outcome of mortality or transfer in sensitivity analyses. The results of this study can guide future research and quality improvement efforts to optimize antibiotic use in NICUs.

Appropriate metrics are a cornerstone of antibiotic stewardship research, yet no metric is uniformly accepted for study in neonatal populations. This has led to heterogeneity in how NICU antibiotic stewardship and quality improvement studies report findings.^2^

AUR and DOT are the most commonly reported metrics in studies of neonatal antibiotic use.^2,4,7,17^ These metrics are calculated in similar fashions and some correlation between the two would be expected. However, it is notable how closely aligned these metrics were in our study. AUR captures both the decision to empirically administer antibiotics (eg, to “rule out sepsis” and to treat for so-called “culture-negative” sepsis) and the imperative to treat specific morbidities (eg, culture-confirmed infection or conditions such as necrotizing enterocolitis.) DOT captures these issues as well as decisions regarding antibiotic choice for each indication. The similarity in dispersion and the similar rate of decline over the study period argues that center-level neonatal antibiotic exposure is driven more by decisions to start and stop antibiotics than by the complexity of prescribed regimens. Given that it may be easier from a data collection standpoint to assess for antibiotics “yes/no” each day (for AUR) versus counting the number of unique antibiotics per day (DOT), AUR may be the optimal measure of antibiotic use in NICUs, but is not necessarily superior in all scenarios,.

ASI can provide additional context focused on the antibiotic spectrum of activity and has previously been studied as a disease-specific metric for treating inpatient pediatric community-acquired pneumonia.^13,18^ One single-center study compared changes in ASI versus DOT among very low birth weight infants with culture-confirmed infections or bowel injuries and found that ASI decreased in response to antibiotic stewardship efforts, while DOT remained unchanged.^15^ Another study examined antibiotic use across three NICUs and found that variations in infection rates and unit guidelines for prescribing antibiotics were reflected in the ASI but not in other standard metrics^14^ Our study did not have available data on infection rates or unit practices, and we observed minimal variability in overall ASI across hundreds of US centers. This consistency suggests a generally uniform national approach to neonatal antibiotic choice. This may be explained by the use of standardized guidance, but may also be related to relatively limited antibiotic options for the neonatal population.^7,19^ Further, a substantial proportion of NICU antibiotic use is attributed to empiric therapy administered for risk of early-onset infection, an indication dominated by the use of the combination of ampicillin and gentamicin.^7^ National guidance for empiric antibiotics for suspected early-onset infections was updated in 2018.^20,21^ ASI declined significantly from 2017 to 2018 and then stabilized, showing only minor variations from 2018 to 2021 (Table 3 and Figure 1). ASI was also lower in Western US centers than in other regions (Supplemental Table 1). Furthermore, in post hoc analysis, late ASI showed greater variability and broader spectrum use than early ASI (Supplemental Figure). Given our findings, ASI may be useful for studying variations and outcomes for specific neonatal indications (such as necrotizing enterocolitis), or among more homogeneous groups of infants, but less useful as a global measure of neonatal antibiotic utilization. ASI may have much more dispersion and utility in non-neonatal settings where there are less uniform antibiotic choices.

Antibiotic stewardship seeks to optimize antibiotic administration choices to benefit patient outcomes. Therefore, metrics utilized in stewardship should align with pertinent outcomes. Risk adjustment for center acuity and patient characteristics is necessary for such comparisons.^2,22,23^ In this study, we were limited by available center- and patient-level data variables and outcomes. None of the three metrics examined was associated with center-level mortality or transfer rates, although AUR and DOT each trended in opposite, albeit non-significant directions and were associated with the combined outcome of mortality or transfer. The association between antibiotic use measures and the composite outcome, but not mortality alone, likely reflects both clinical and statistical factors. Inter-NICU transfer may capture serious illness not reflected in mortality alone, particularly in centers lacking subspecialty services. Additionally, the higher frequency of the composite outcome increased power to detect associations, suggesting it may serve as a more sensitive, though indirect, marker of adverse events.

The strengths of this study were the large sample size and the inclusion of multiple years of data, which allowed for a robust evaluation of trends over time across hundreds of US NICUs. However, limitations must also be considered. Our analysis was conducted at the center level, and there was a lack of granular patient-level data and detailed short- and long-term outcomes. Future studies should further delineate the relationships between metrics and appropriateness of antibiotic use in NICUs and outcomes beyond mortality or transfer. We only assessed three metrics and did not include more recently proposed metrics, including the neonatal standardized antimicrobial administration ratios (SAAR).^24–26^ Details on center antibiotic prescription guidelines and microbiology laboratory testing protocols, patient microbiology and laboratory results, and appropriateness of antibiotic choice based on an indication were not available. An important limitation of DOT that we could not address in this study is that this metric may theoretically encourage broader-spectrum monotherapy.^2^ The impact of the COVID-19 pandemic on antibiotic utilization during the study period was not assessed.

## Conclusion

Antibiotic use in US NICUs decreased from 2017 to 2021, as measured by three metrics. DOT and AUR are comparable measures of antibiotic consumption.

ASI provides additional insight into the breadth of antibiotic coverage but may be limited by the relatively low variability inherent in the NICU, particularly in the first three days after birth. None of the metrics were significantly associated with center-level mortality. These findings reinforce the importance of assessing the utility of multiple metrics in neonatal antibiotic stewardship efforts and highlight the need for risk-adjusted comparisons to ensure meaningful benchmarking and quality improvement initiatives in NICUs.

## Supplementary material

The supplementary material for this article can be found at https://doi.org/10.1017/ice.2025.10233

## Supporting information

Zevallos Barboza et al. supplementary material 1 Zevallos Barboza et al. supplementary material

Zevallos Barboza et al. supplementary material 2 Zevallos Barboza et al. supplementary material

## Abbreviations

ASI: antibiotic spectrum index

AUR: antibiotic use rate

DOT: days of therapy

NICU: neonatal intensive care unit

PD: patient days

SAAR: standardized antimicrobial administration ratio

VLBW: very low birth weight
