# Supplementary material for: Comparison of metrics of neonatal intensive care unit antibiotic use
Source: Infect Control Hosp Epidemiol. 2025 Aug 19;46(9):903–9. doi: 10.1017/ice.2025.10233 (PMC12616220; doi:10.1017/ice.2025.10233)
Supplement: Zevallos Barboza et al. supplementary material 1 — Zevallos Barboza et al. supplementary material [file S0899823X2510233Xsup001.docx]

**Supplemental Table 1**: Comparison of metrics by center characteristics

|  | **Teaching (n=103)** | | **Non-teaching (n=169)** | | **p-value*** |
| --- | --- | --- | --- | --- | --- |
| AUR | 126.3 (91.1 – 170.7) | | 123.4 (90.1 – 166.5) | | 0.64 |
| DOT | 214.1 (151.5 – 275.5) | | 204.0 (151.9 – 287.1) | | 0.97 |
| ASI | 5883.7 (5472.3 – 6128.5) | | 5772.7 (5393.0 – 6003.3) | | 0.03 |
|  | **Rural**  **(n=46)** | | **Urban**  **(n=226)** | |  |
| AUR | 143.6 (91.2 – 181.1) | | 122.0 (90.5 – 164.8) | | 0.16 |
| DOT | 261.1 (154.0 – 317.9) | | 201.6 (151.5 – 275.1) | | 0.08 |
| ASI | 5863.7 (5590.4 – 6114.1) | | 5799.8 (5415.5 – 6039.7) | | 0.34 |
|  | **Midwest (n=71)** | **Northeast (n=32)** | **South (n=127)** | **West (n=42)** |  |
| AUR | 113.9 (87.3 - 165.7) | 138.5 (94.2 - 159.5) | 128.1 (92.5 - 176.5) | 120.4 (87.2 - 154.2) | 0.68 |
| DOT | 196.8 (151.9 - 277.2) | 229.8 (155.2 - 274.7) | 216.3 (152.2 - 303.2) | 196.1 (139.4 - 259.3) | 0.67 |
| ASI | 5881.3 (5569.7 - 6078.1) | 5875.2 (5658.0 – 6094.0) | 5803.7 (5463 - 6056.7) | 5506.6 (5065.1 - 5957.5) | 0.04 |

Footnote:

* Mann-Whitney U test was used to compare the medians and determine if there is a significant difference between the groups

**Supplemental Table 2:** Associations between metrics and crude center death rates, transfer rates, and death or transfer rates adjusting for available center-level variables

| **Death rate (Deaths per 1000 NICU infants) ^a^** | | | |
| --- | --- | --- | --- |
| Metric | Coefficient estimate ^b^ | 95% CI | p-value |
| AUR | -0.006 | (-0.01) - (0.002) | 0.12 |
| DOT | -0.004 | (-0.009) - (0.00005) | 0.05 |
| ASI | 0.0004 | (-0.0002) - (0.001) | 0.14 |
| **Transfer rate (Transfers per 1000 NICU infants) ^a^** | | | |
| Metric | Coefficient estimate ^b^ | 95% CI | p-value |
| AUR | 0.2 | (0.1) - (0.3) | <0.001 |
| DOT | 0.1 | (0.08) - (0.2) | <0.001 |
| ASI | 0.004 | (-0.0003) - (0.01) | 0.07 |
| **Death or transfer rate (Deaths or transfers per 1000 NICU infants) ^a^** | | | |
| Metric | Coefficient estimate ^b^ | 95% CI | p-value |
| AUR | 0.2 | (0.1) - (0.3) | <0.001 |
| DOT | 0.1 | (0.08) - (0.15) | <0.001 |
| ASI | 0.005 | (0.0002) - (0.01) | 0.04 |

Footnotes:

^a^ A mixed-effects model was adjusted for geographical region, setting, teaching status, number of beds, and year.

^b^ Coefficient estimate reports the change in center rate (occurrence per 1000 NICU infants) for a unit increase in the respective metric after adjusting for the center-level variables.

**Supplemental Figure**: ASI values stratified by early (first 3 days) and late (after 3 days) administration timing

Footnote: ASI (antibiotic spectrum index)

**Left Panel:** Distribution of center-level ASI values derived from NICU encounter data spanning 2017–2021. Data are stratified by the timing of antibiotic administration: the *early* ASI (antibiotics administered within the first three days of hospital admission) versus *late* ASI (antibiotics administered after the first three days).

**Right Panel:** Density curves illustrating the overall distribution of ASI values for both groups, highlighting differences in relative frequency and dispersion.

A Mann-Whitney U test confirmed a statistically significant difference in the median between the two groups (p < 0.001).

**Appendix A**: Antibiotic Spectrum Index

| **ANTIBIOTIC** | **ASI** |
| --- | --- |
| Amikacin | 6 |
| Amoxicillin-clavulanate | 6 |
| Amoxicillin | 2 |
| Ampicillin | 2 |
| Ampicillin-sulbactam | 6 |
| Azithromycin | 4 |
| Aztreonam | 3 |
| Cefadroxil* | 3 |
| Cefazolin | 3 |
| Cefdinir | 3 |
| Cefepime | 6 |
| Cefixime | 3 |
| Cefotaxime | 5 |
| Cefotetan* | 3 |
| Cefoxitin | 5 |
| Cefpodoxime | 3 |
| Cefprozil | 4 |
| Ceftaroline | 8 |
| Ceftazidime | 4 |
| Ceftazidime-avibactam* | 7 |
| Ceftriaxone | 5 |
| Cefuroxime | 4 |
| Cephalexin | 2 |
| Ciprofloxacin | 8 |
| Clindamycin | 4 |
| Cloxacillin* | 1 |
| Colistimethate | 5 |
| Daptomycin | 5 |
| Doxycycline | 5 |
| Ertapenem | 9 |
| Erythromycin | 2 |
| Erythromycin-sulfisoxazole* | 2 |
| Gentamicin | 5 |
| Imipenem* | 11 |
| Levofloxacin | 9 |
| Linezolid | 6 |
| Meropenem | 10 |
| Meropenem-vaborbactam | 11 |
| Metronidazole | 2 |
| Moxifloxacin | 10 |
| Nafcillin* | 1 |
| Oxacillin | 1 |
| Penicillin | 2 |
| Piperacillin | 4 |
| Piperacillin-tazobactam | 8 |
| Quinupristin-dalfopristin* | 5 |
| Rifampin | 3 |
| Trimethoprim-sulfamethoxazole | 4 |
| Tobramycin | 5 |
| Vancomycin | 5 |

Footnotes: (ASI) Antibiotic Spectrum Index, adapted from Gerber *et al*. Drugs that were excluded: atovaquone, dicloxacillin, chloramphenicol, clarithromycin, imipenem-cilastatin, minocycline, neomycin, oxytetracycline, pentamidine, sulfadiazine, telavancin, ticarcillin-clavulanate, tigecycline.

* Denotes antibiotics which were not assigned an ASI point value in previous study; points were assigned based on closeness to drugs of the same class or activity spectrum by a pediatric infectious diseases physician (JSG).

**Appendix B**: ICD-10 and APR-DRG codes used to define very-low birth weight and gestational age

| **ICD-10 Codes** | |
| --- | --- |
| **Very-low birth weight (<1500g)** | |
| P05.01 | Newborn light for gestational age, less than 500g |
| P05.11 | Newborn small for gestational age, less than 500g |
| P07.01 | Extremely low birth weight newborn, less than 500g |
| P05.02 | Newborn light for gestational age, 500-749g |
| P05.12 | Newborn small for gestational age, 500-749g |
| P07.02 | Extremely low birth weight newborn, 500-749g |
| P05.03 | Newborn light for gestational age, 750-999g |
| P05.13 | Newborn small for gestational age, 750-999g |
| P07.03 | Extremely low birth weight newborn, 750-999g |
| P05.04 | Newborn light for gestational age, 1000-1249g |
| P05.14 | Newborn small for gestational age, 1000-1249g |
| P07.14 | Extremely low birth weight newborn, 1000-1249g |
| P05.05 | Newborn light for gestational age, 1250-1499g |
| P05.15 | Newborn small for gestational age, 1250-1499g |
| P07.15 | Extremely low birth weight newborn, 1250-1499g |
| **Gestational age <29 weeks** | |
| P07.20 | Extreme immaturity of newborn, unspecified weeks of gestation |
| P07.21 | Extreme immaturity of newborn, gestational age less than 23 completed weeks |
| P07.22 | Extreme immaturity of newborn, gestational age 23 completed weeks |
| P07.23 | Extreme immaturity of newborn, gestational age 24 completed weeks |
| P07.24 | Extreme immaturity of newborn, gestational age 25 completed weeks |
| P07.25 | Extreme immaturity of newborn, gestational age 26 completed weeks |
| P07.26 | Extreme immaturity of newborn, gestational age 27 completed weeks |
| P07.31 | Preterm newborn, gestational age 28 completed weeks |

| **APR-DRG Codes** | |
| --- | --- |
| **Very-low birth weight (<1500g)** | |
| 588 | Neonate birth weight <1500g w major procedure |
| 589 | Neonate birth weight <500g |
| 591 | Neonate birth weight 500-749g w/o major procedure |
| 593 | Neonate birth weight 750-999g w/o major procedure |
| 602 | Neonate birth weight 1000-1249g w resp dist synd/oth maj resp or maj anom |
| 603 | Neonate birth weight 1000-1249g w or w/o other significant condition |
| 607 | Neonate birth weight 1250-1499g w resp dist synd/oth maj resp or maj anom |
| 608 | Neonate birth weight 1250-1499g w or w/o other significant condition |
| 588 | Newborn small for gestational age, 750-999g |
