# Supplementary figures and images for: Comparison of metrics of neonatal intensive care unit antibiotic use
Source: Infect Control Hosp Epidemiol. 2025 Aug 19;46(9):903–9. doi: 10.1017/ice.2025.10233 (PMC12616220; doi:10.1017/ice.2025.10233)

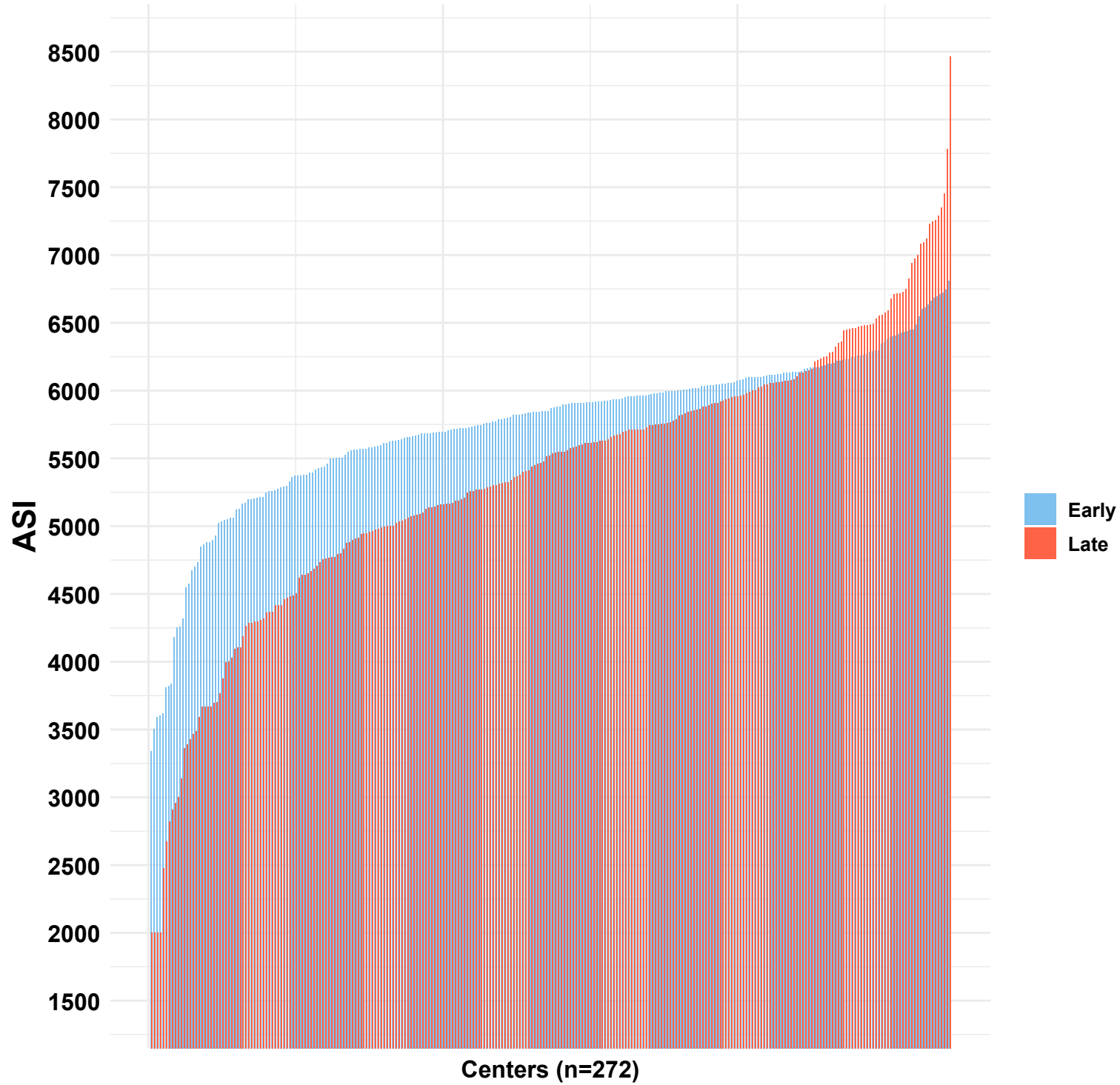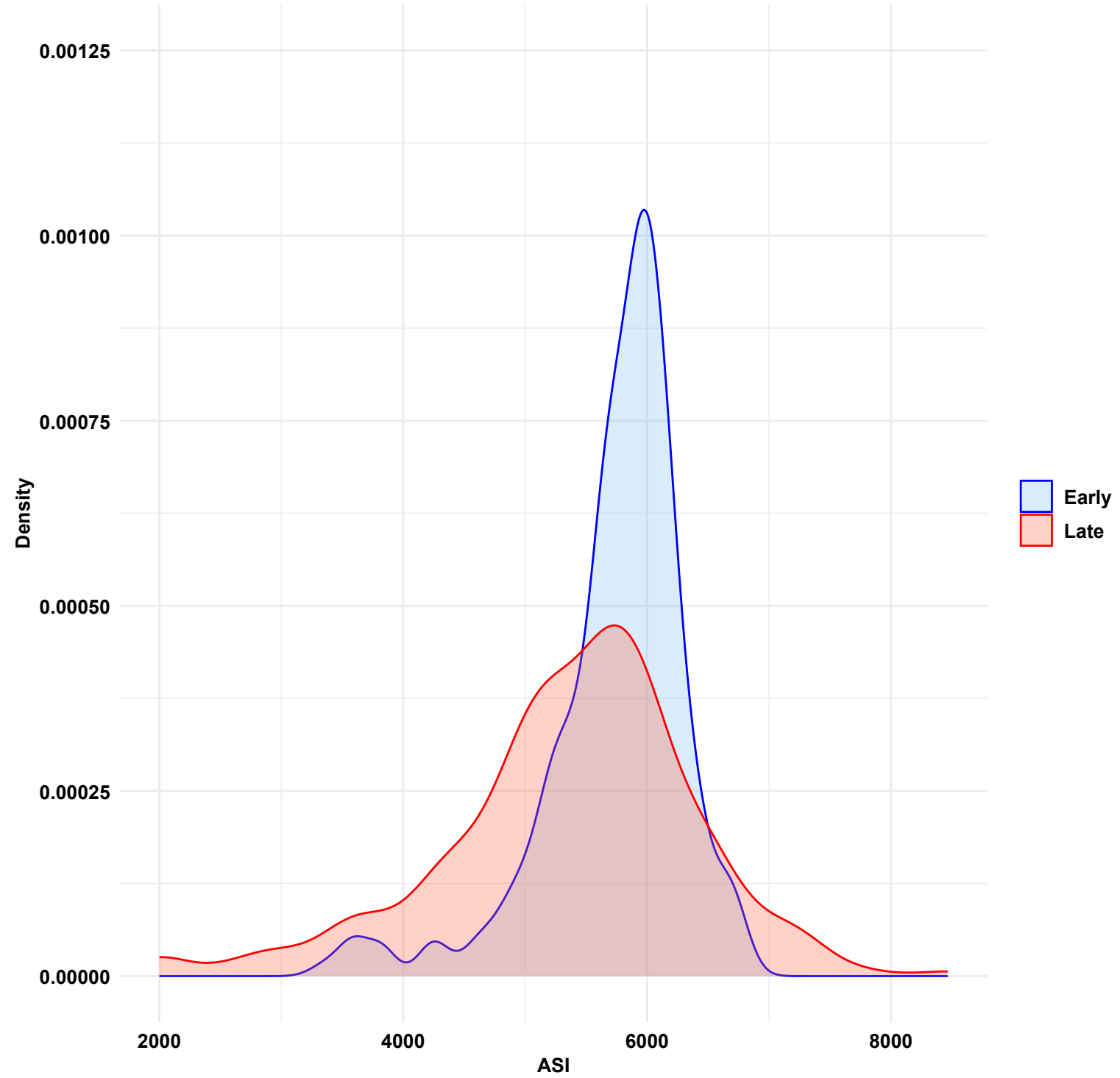

Supplement: Zevallos Barboza et al. supplementary material 2 — Zevallos Barboza et al. supplementary material [file S0899823X2510233Xsup002.pdf]
